# Comparing Two Intestinal Porcine Epithelial Cell Lines (IPECs): Morphological Differentiation, Function and Metabolism

**DOI:** 10.1371/journal.pone.0132323

**Published:** 2015-07-06

**Authors:** Constanze Nossol, Anicò Barta-Böszörményi, Stefan Kahlert, Werner Zuschratter, Heidi Faber-Zuschratter, Nicole Reinhardt, Siriluk Ponsuksili, Klaus Wimmers, Anne-Kathrin Diesing, Hermann-Josef Rothkötter

**Affiliations:** 1 Institute of Anatomy, Otto-von-Guericke University Magdeburg, 39120, Magdeburg, Germany; 2 Leibniz Institute of Neurobiology, 39120, Magdeburg, Germany; 3 Leibniz Institute of Farm Animal Biology (FBN) Dummerstorf, 18196, Dummerstorf, Germany; INRA, UR1282, FRANCE

## Abstract

The pig shows genetical and physiological resemblance to human, which predestines it as an experimental animal model especially for mucosal physiology. Therefore, the intestinal epithelial cell lines 1 and J2 (IPEC-1, IPEC-J2) - spontaneously immortalised cell lines from the porcine intestine - are important tools for studying intestinal function. A microarray (GeneChip Porcine Genome Array) was performed to compare the genome wide gene expression of IPECs. Different significantly up-regulated pathways were identified, like “lysosome”, “pathways in cancer”, “regulation of actin cytoskeleton” and “oxidative phosphorylation” in IPEC-J2 in comparison to IPEC-1. On the other hand, “spliceosome”, “ribosome”, “RNA-degradation” and “tight junction” are significantly down-regulated pathways in IPEC-J2 in comparison to IPEC-1. Examined pathways were followed up by functional analyses. ATP-, oxygen, glucose and lactate-measurement provide evidence for up-regulation of oxidative phosphorylation in IPEC-J2. These cells seem to be more active in their metabolism than IPEC-1 cells due to a significant higher ATP-content as well as a higher O_2_- and glucose-consumption. The down-regulated pathway “ribosome” was followed up by measurement of RNA- and protein content. In summary, IPEC-J2 is a morphologically and functionally more differentiated cell line in comparison to IPEC-1. In addition, IPEC-J2 cells are a preferential tool for *in vitro* studies with the focus on metabolism.

## Introduction

There is increasing need for suitable enterocytic cell cultures of the jejunum and ileum. Because intestinal disorders are a significant cause of morbidity and mortality in the population world wide, the knowledge of the molecular and biological epithelial cell functions is therefore of special importance. The use of the omnivorous pig as a model to mimic the human intestinal barrier function is given through the anatomical and physiological similarities. The abdominal organs like stomach, spleen, bile duct system, small intestine, kidney and bladder found in pigs are basically the same found in human [1]. Furthermore, similar to humans, *Firmicutes* and *Bacteroidetes* phyla [2] colonise the gut of pigs. Therefore, the pig model can be used in the areas of amino acid metabolism, total parental nutrition and common bacterial as well as viral infections (e.g. rotavirus). Several methods have been used to establish cell monolayers comparable to the polarised gut columnar epithelium. Cultivation of permanent cell lines on permeable support membranes allows the access to both the apical and basolateral compartment of the monolayer. In comparison to the use of short-term Ussing chamber experiments (up to 3 hours), these cell cultures can be used over a period of days for studies in cellular protein and nutrient transport, digestion, pharmacological regulation and microbial exposition.

However, the number of cells available for these cultivation methods is limited. The human intestinal Caco-2 cell line has often been used to study the differentiation of epithelial cells [3], enzymes location within the brush border [4], expression of nutrient transporters [5] and adhesion molecules [6]. However, the Caco-2 cell line was generated from human colonic cancer and despite the described epithelial like structure and function, Caco-2 cells still harbour properties derived from the original colonic cancer source. For a more detailed study of the epithelial function a cell system is necessary to compare the cell culture data with the physiological situation *in vivo*.

In the recent years the intestinal porcine epithelial cell lines IPEC-1 and IPEC-J2, derived from pig small intestine, have been used especially for epithelial microbiological exposition [7]. Their morphology and growth on different basolateral support media has been studied in detail [8]. The IPEC-1 and IPEC-J2 cells are both non-transformed and had their origin in piglets less than 12 hours old [9]. For these cell lines there is limited knowledge about the constitutive “baseline” morphology, structure and metabolism—and the underlying gene expression.

For better understanding of the IPEC-cell lines in terms of growth, metabolism and suitability for microbial and drug testing, in the present study both IPEC-1 and IPEC-J2 were analysed for gene expression (microarray analysis and qPCR), morphology, metabolism as well as cytoskeleton and tight junction structure. Based on microarray analysis, different genes were selected for brush border, structure proteins, cell cycle, metabolism and tight junction proteins, and the protein content in the cells was determined. The final aim of this study was to characterise these cell lines to provide a well-defined *in vitro* model comparable with intestinal physiology.

## Material and Methods

### Cell culture and transepithelial electrical resistance (TEER) measurement

Intestinal porcine epithelial cells (IPEC-1 ACC 701 and IPEC-J2 ACC 705; [10], Leibniz Institute DSMZ—German Collection of Microorganisms and Cell Cultures, Braunschweig, Germany) were regularly tested and found to be free of mycoplasma contamination (Venor GeM Mycoplasma Detection Kit; Minerva Biolabs, Berlin, Germany). In all experiments, cells were seeded with a density of 0.88*10^5^/cm^2^ on permeable support (ThinCerts; pore size: 1 μm; polyester; Greiner bio-one, Germany). DMEM/HAMs F12 supplemented with 5% foetal calf serum (FCS), 5 ml/500 mL cell culture medium, 16 mM 4-(2-hydroxyethyl)-1-piperazineethansulfonic acid (HEPES) (all PAN-Biotech, Aidenbach, Germany) and 5 ng/mL epidermal growth factor (EGF, Biochrome, Berlin, Germany) was used as culture medium. Cells grew at 39°C in an atmosphere of 5% CO_2_ and 95% relative humidity. The transepithelial electrical resistance (TEER) was measured on day 7, 8, 9 and 10 of culture using a Millicell-TERS-electrode (Millipore, Berlin, Germany). Prior to the measurement, the electrode was washed in 70% alcohol and ampuwa (Brand, Melsungen, Germany), then the electrode was washed in pre-warmed medium and the TEER was determined. The same order of alcohol, ampuwa and pre-warmed medium was used to clean the electrode between the measurements. For the analysis of p53 cells were treated with p53 activator (10 μM; Merck, Darmstadt, Germany) for 24 h.

### Anchorage independence growth

Soft agar assays (n = 3/cell line) consists of “Feeder agar” (0.5% agar) and “Soft agar” (0.33%; Sigma, Hamburg, Germany). Both–“Feeder agar” and “Soft agar”–depend on DMEM/HAMs F12 supplemented with 5% FCS, penicillin (100 units/mL) and streptomycin (100 μg/mL). “Feeder agar” and/or “Soft agar” were supplemented with 5 ng/mL EGF and 5 mL/500 mL ITS (both: PAN-Biotech, Aidenbach, Germany) to check for possible effects of the supplementation. The “Feeder agar” was poured into a 25 cm^2^-culture flask, allowed to solidify and built the basis for the “Soft agar”. On the “Feeder agar”, 1*10^5^ cells in 1.5 mL 0.33% “Soft agar” were seeded. Caco-2 cells (ATCC HTB37, a kind gift from Dr. Schierack, Institute of Microbiology and Animal Epidemic, Charitè, Berlin [3]) were used as positive control. The cultures were maintained at 39°C in 5% CO_2_ for up to 3 weeks.

### RNA isolation

TRIzol reagent (Invitrogen, Darmstadt, Germany) was added to samples of IPECs as described in the manufacturer’s protocol. On day 10, cells were scraped from the support and lysed. In the next step, chloroform was added to the cell suspension and RNA recovered from the aqueous phase by precipitation with isopropyl alcohol. The dried pellet was dissolved in diethylpyrocarbonate (DEPC) water (Roche, Basel, Switzerland). After DNaseI treatment the RNA was cleaned up with the RNeasy Kit (Qiagen, Venlo, The Netherlands). The quantity of RNA was determined using the NanoDrop ND-1000 spectrophotometer (Peqlab, Erlangen, Germany) and the integrity was checked by running 1 μg of RNA on a 1% agarose gel. The RNA samples were stored at -80°C until processing.

### Microarray and data analysis

The microarray was performed in three independent experiments. Briefly, Gene Chip Porcine Genome Array (Affymetrix, High Wycombe, UK) was used, loaded with 500 ng RNA and analysed by provided software (Affymetrix GCOS 1.3) using global scaling to a target signal of 500. The array analyses were performed as described in Diesing et al. (2012) [8]. The microarray data were analysed in 3 steps: (A) quality control of all arrays, (B) preprocessing of all arrays (background correction, normalization, summary measures for probe sets), and (C) identification of differently expressed genes.

Quality control, data preprocessing and statistical analysis were performed using the R statistical language (Bioconductor Packages, (http://www.bioconductor.org/), employing methods described before [11].

In the next step, Affymetrix IDs were mapped to the corresponding gene symbols based on the assignments available from the Ensembl database (http://www.ensembl.org). Mean values were calculated over all corresponding Affymetrix IDs. A t-test was used to assess statistical significance of differentially expressed genes (p < 0.05) between the cell lines. The resulting lists of up and down-regulated genes was used to identify functional pathways in both cell lines using the DAVID Bioinformatic resources [12]. Biological pathways were designated according to the Kyoto Encyclopedia of Genes and Genome (KEGG) database (http://www.genome.jp/kegg/). Our microarray data are deposited under the Gene Expression Omnibus (GEO) with the acc.no. GSE67407 (http://www.ncbi.nlm.nih.gov/geo/query/acc.cgi?acc=GSE67407).

### Real Time Quantitative PCR

The qPCR was performed as described in Nossol et al. (2011) [13]. The Qubit RNA Assay Kit was used to measure RNA-content (life technologies, Darmstadt, Germany). The used primer pairs are shown in Table 1. The analysis consisted of at least 5 independent experiments. Each experimental sample was assayed using triplicates for each primer pair. The ΔΔCT-method was used for calculation of differences in the expression profiles. The relative difference of the expression between both cell lines, which was normalised to the reference genes, β-actin and 18S, resulted in the set phrase: ratio = 2^-ΔΔCT^. No marked differences were found between both reference genes. Samples were tested on”Normal Distribution” with a “Kolmogorov-Smirnov-Test”. In the case of “Normal Distribution” a “T-Test” was used to examine the differences between the genes. If no “Normal Distribution” was observed, a “Welch-Test” was done (SPSS 22).

**Table 1:** qPCR—used primer sequences.

name	function	sequence 5‘ → 3‘ primer left	sequence 5‘ → 3‘ primer right	efficiency [%]	temperature [°C]
GAPDH	glycerinaldehyde-3-phos-dehydrogenase	acccagaagactgtgga	ttgagctcagggatgacctt	101.8	59.5
actin	cytoskeleton	gatgagattggcatggcttt	caccttcaccgttccagttt	99.3	58.3
VIL-1	Villin-1	caccatgaccaaactgaacg	tcgaagaagctgccataggt	98.4	54
MUC4	mucin-4	gctgacaggaagaggccata	ccccttcaactctggtgtgt	100	54
TLR4	Toll-like-rezeptor 4	ctggagacgactcaagaagc	agtgaaggctgttgtatcatgc	97.4	60.6
OCLN	occludin (TJ)	tgacactctaggcaatcaggtc	gggcccaatttccttatctg	98.5	54
VIL-2	villin-2 (ezrin)	cagtcgacgaaatcctgtga	ggtgcaggtccaacaaggta	97.5	57.1
Espin	espin	agaagcggaaagaggaggag	acttagcgatgtcccccttc	97.2	61.6
HIF1a	hypoxia inducible factor	tgccagaacctcctgtaacc	atgtacgtggggaggagatg	94.8	54
p53	p53	cgaactggcatgaaaat	agaagggacaaaggacgaca	105.8	56.2
CLDN1	claudin-1	ccagtgaagagagcctgacc	tgatgaggtgcagaagatgc	98.5	60.6
CLDN3	claudin-3	gtccatgggcctggagat	gatctgcgctgtgataatgc	99.5	58.7
CLDN7	claudin-7	ctcactcccaggacaagagc	tttgtgcgttgatagcttgc	99.3	60.6
BAD	BCL-2-antagonist	cttgcaaaaagagccgtttc	ttaagggcggaaaaacttca	98.3	57.6
BCL-2	apoptose-regulator	atttagcctcttgcctgtgg	gcagtttgaggctgcttttt	96.9	54
BAX	apoptose-regulator	ggtcgcgcttttctactttg	cgatctcgaaggaagtccag	99.6	60.6
PDH	pyruvat-dehydrogenase	acccgatcatgcttctcaag	tagcaaactgtgcagcatcc	96.7	54
SDH	succinat-dehydrogenase	actggatgggctgtacgagt	gtcgatcatccagcgatagg	96.7	55.7
COX5B	Cytochrome c oxidase subunit 5B	ggagagggaggtcatgatgg	ccactatccgcttgttggtg	100.2	61.2
18S	Ribosomal RNA	gcaattattccccatgaacg	ggcctcactaaaccatccaa	103.9	56.5
RPL10a	Ribosomal protein	tgcctttttggcttcagaat	actttggccaccatgttctc	98.7	54.7
Cyc 1	Cytochrome C	ctaccatgtcccaggtagcc	aaagcaagcccatcatcatc	96.7	54.7

### Western blot

Western blot analyses (n = 3) were performed as described in Nossol et al. (2011) [13], and the Qubit Protein Assay Kit was used to measure the protein content (Life Technologies, Darmstadt, Germany). The samples were analysed with the Qubit 2.0 Fluorometer following the manufacturer’s instructions (Invitrogen, Darmstadt, Germany). Samples (40 μg) were loaded on sodium dodecyl sulfate (SDS) polyacrylamide gel in parallel with the pre-stained protein ladder (SM1811, 10–250 kD; Fermentas, Darmstadt, Germany) and subsequently transferred to PVDF membrane by semi-dry electroblotting. Different antibodies were used: rabbit anti-human ZO-1 (*Zonula occludens protein* 1, 1:500), mouse anti-human Occludin (1:2500; both: Invitrogen, Germany), rabbit anti-human claudin-3, mouse anti-human claudin-4 (both: 1:1000, Invitrogen, Darmstadt, Germany), mouse anti-human CK18 (Cytokeratin 18, 1:5000; Cy90; Sigma-Aldrich, Hamburg, Germany), mouse anti-human p53 (1:500, clone PAb 240, Kamiya Biomedical Company, Seattle, USA), mouse anti-human β-actin (1:40000, Cell signalling, Leiden, The Netherlands), rabbit anti-mouse BAD (BCL-2-associated agonist of cell death, 1:100; Abcam, Cambridge, UK), rabbit anti-human BAX (BCL-2-associated X protein, 1:100; Abnova, Taoyuan City, Taiwan), mouse anti-human BCL-X (apoptosis regulator, 1:200; Invitrogen, Darmstadt, Germany), mouse anti-human ezrin (1:5000, Abcam, Cambridge, UK); mouse anti-chicken villin (1:2500, Acris, Herford, Germany); goat anti-human RPL10a (ribosomal protein 10a, 1:1000, LS BioSeattle, USA) and rabbit anti-human COX5B (cytochrome C oxidase subunit 5B, 1:1000, WuXi App Tec, Shanghai, China). The secondary antibody was purchased with the BM Chemiluminescence Western Blotting Kit mouse/rabbit (Roche, Basel, Switzerland). Blots were analysed on an Alpha-Ease FC Imaging System (Alpha Innotech, Kasendorf, Germany).

### Immunofluorescence and confocal microscopy

IPEC monolayers were fixed and stained as described in Nossol et al. (2011) [13]. Briefly, for the analysis of the tight junctions and cytoskeleton different primary antibodies were used: rabbit anti-human ZO-1, rabbit anti-human claudin-3, mouse anti-human claudin-4 (all: 1:100, Invitrogen, Darmstadt, Germany), mouse anti-human β-catenin (1:100, Abcam, Cambridge, UK) and mouse anti-porcine CK18 (1:5000, Sigma-Aldrich, Hamburg, Germany). Phalloidin-PromoFluor-488 was used to stain actin in the monolayers (1:40; Promo Kine, Heidelberg, Germany). In every experiment a secondary antibody control was examined to check for unspecific binding. Images shown are representative of at least three experiments, with multiple areas imaged per slide. Confocal imaging was performed by using a commercial laserscanning microscope Leica TCS SP5 (Leica Microsystems, Wetzlar, Germany). Briefly, the system comprises an upright microscope (DM 6000 CFS with a Tandem Scanning System (SP5), Acousto-Optical Tunable Filters (AOTF) and an Acousto-Optical Beam Splitter (AOBS)) equipped with a Diode 405 nm laser, an Argon laser (wavelengths: 458, 476 488, 496, 514 nm), a DPSS 561 laser and HeNe 594 nm and 633 nm lasers. Confocal images were taken by scanning the focused laser beam with a galvo-mirror through a HCX PL APO CS 63 x NA 1.4 oil objective (Leica Microsystems, Wetzlar, Germany) at zoom factor 5 across the specimen. Unidirectional scans were recorded at scan speeds of 400 or 700 Hz with line averaging of 3. Confocal image stacks consisting of 33 to 75 focal planes with an axial step size of 0.21 μm (total depths between 7 and 15 μm) were digitized with 8 bit depth at 1024 x 1024 pixel resolution resulting in a x, y, z voxel size of 48.9 nm x 48.9 nm x 209.8 nm. Fluorescence signals of the different dyes were detected sequentially (between laser lines) by photomultiplier tubes (ch 2 and 3) or GaAsP hybrid detectors (ch1) within three spectral regions (501–542 nm for Alexa 488 (ch1), 569–611 nm for Texas_RED_ (ch 2) and 415–457 nm for DAPI (ch 3)). After scanning images were processed contrast and brightness levels of individual channels using either ImageJ (National Institutes of Health, USA) or Photoshop CS 5 (Adobe. System Inc., San Jose, USA),

### Transmission electron microscopy

Samples were treated and analysed as described in Nossol et al. (2011) [13]. The semi-thin sections stained with toluidine blue were used to measure the layer thickness. For semi-quantitative analysis 4 grids (1 grid is equivalent to 2 mm cell culture section) were analysed per time (day 10 and day 31 of cultivation) and cell line. 10 pictures/time/cell line were used to determine cell number/10 μm and projected to 8 mm general cell culture section. In the next step, 10 pictures/time/cell line were chosen to measure microvilli number/10 μm. The same pictures were taken to examine the microvilli length in both cell lines at the two different times.

### ATP-Assay

Cells were seeded on 12-well-ThinCerts for 10 days. On day 9, cells were treated with Carbonylcyanid-4-trifluormethoxyphenylhydrazon (FCCP,5 μM, Sigma-Aldrich, Hamburg, Germany) for 24 h. On day 10, media was removed and a boiling hot puffer (300 μl/well; 100 mM TRIS; 4 mM EDTA; pH = 7.75, Roth, Karlsruhe, Germany) was added to the cells. In the next step, cells were scraped off the membrane with a cell scraper. The cell suspension was transferred into a tube, incubated for 2 min at 100°C, centrifuged at 1000xg for 60 s. Supernatants were pipetted into a white 96-microplate (50 μl/well; triplicates; Greiner bioone; Frickenhausen, Germany). Samples kept on ice until measurement. An ATP-standard curve was prepared after manufacturer’s instructions (5 readings within 5 min; 25°C; ATP Bioluminescence Assay Kit CLS II; Roche, Basel, Switzerland).

### Oxygen measurement

The oxygen uptake was measured with a Microx TX3 (PreSens, Regensburg, Germany). Microx TX3 is a micro fiber optic oxygen meter with a microsensor based on a 140 μm optical fiber. The sensor was calibrated after manufacturer’s instructions (manual; 2-point-calibration). The oxygen content in the medium without cells and the oxygen content in the medium (apical from the cells was measured over 10 minutes in glass bottom dishes (Mat Tek, Ashland, USA) and ThinCerts). The oxygen uptake is given due to the difference between both values (sample value—blank value).

### Lactate and glucose measurement

Lactate and glucose content of both cell lines was analysed on day 10 of cultivation. Therefore, apical and basolateral supernatants were collected of both cell lines. Medium was changed 72 h before and medium without cells was used as blank. All samples were stored on ice and immediately measured with a Roche/Hitachi Cobas c system (Roche, Basel, Switzerland). The difference between blank and sample represents the glucose consumption respectively lactate production of the cell.

### Statistical analysis

Results of the ATP, Glucose and Lactate measurement, protein and RNA-content are expressed as means and standard deviation (S.D.) of at least 5 independent experiments. Asterisks indicate significant differences from IPEC-1 (*p≤0.05; **p≤0.01; ***p≤0.001). The analysis of the ATP-content of both cell lines was examined using an univariate analysis of variance (*p≤0.05; **p≤0.01; ***p≤0.001).

## Results

Both cell lines were cultured on membranes for 10 days. The analysis of the gene expression profile of IPEC-1 and IPEC-J2 aimed to generate a list of genes differentially expressed in both cell lines. 14 746 genes were analysed and 5 787 varied significantly. We observed 2 631 genes up-regulated and 3 156 genes down-regulated in IPEC-J2 in comparison to IPEC-1. Up- and down-regulated KEGG (Kyoto encyclopedia of genes and genome) pathways in IPEC-J2 in comparison to IPEC-1 are shown in Table 2. Different pathways were analysed in the next step.

**Table 2:** Different regulated pathways. Different pathways are shown which are significantly up (+) or down (-) regulated in IPEC-J2 in comparison to IPEC-1.

	**significant up-regulated pathways**	**in IPEC-J2**
1	lysosome	+
2	focal adhesion	+
3	pathways in cancer	+
4	N-Glycan biosynthesis	+
5	endocytosis	+
6	glycosphingolipid	+
7	regulation of actin-cytoskeleton	+
8	glycosaminoglycan degradation	+
9	oxidative phosphorylation	+
10	ECM-receptor interaction	+
	**significant down-regulated pathways**	**in IPEC-J2**
1	spliceosome	-
2	Ribosome	-
3	ubiquitin mediated proteolysis	-
4	protein export	-
5	RNA degradation	-
6	Wnt signaling pathway	-
7	tight junctions	-

**Fig 1:**
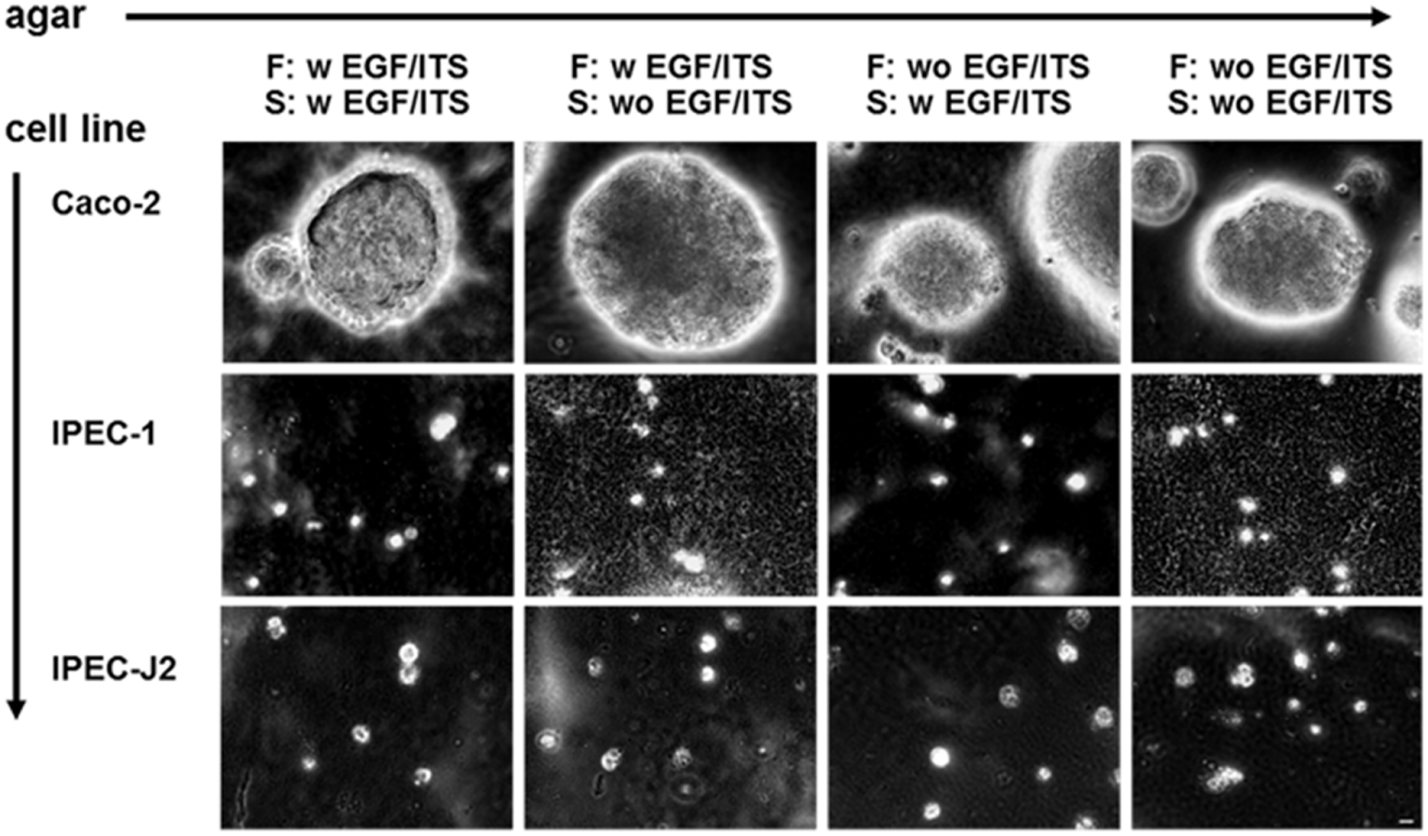
Anchorage independent growth. Both cell lines were seeded in “Soft agar” (S, 0.33%) on “Feeder agar” (F, 0.5%) with (w) or without (wo) the application of EGF and ITS. Caco-2 cells were used as positive control. No anchorage independent growth was detected in IPEC-1 and IPEC-J2. On the other hand, Caco-2 showed an anchorage independent growth which did not depend on the additives EGF and ITS (bar = 20 μm). The results represent at least three independent experiments (n = 3).

IPEC-1 and IPEC-J2 are known to be non-transformed cell lines derived from normal intestinal cells. Typically, non-transformed epithelial cells do not grow in soft agar without solid cultivation surface. The anchorage independent growth was here used as a functional marker of tumorgenicity and compared with Caco-2 cells as a positive control. Caco-2 cells produced colonies within the agar independent of the additives of “feeder- and soft-agar”. In comparison to Caco-2, IPEC-1 and IPEC-J2 are non-infiltrating cells (Fig 1). After 3 weeks, only single cells and no colonies were detected. The supplementation of EGF and ITS (important growth factors for all three cell lines) to “feeder- and soft-agar” had no effects on the growth of IPECs.

**Fig 2:**
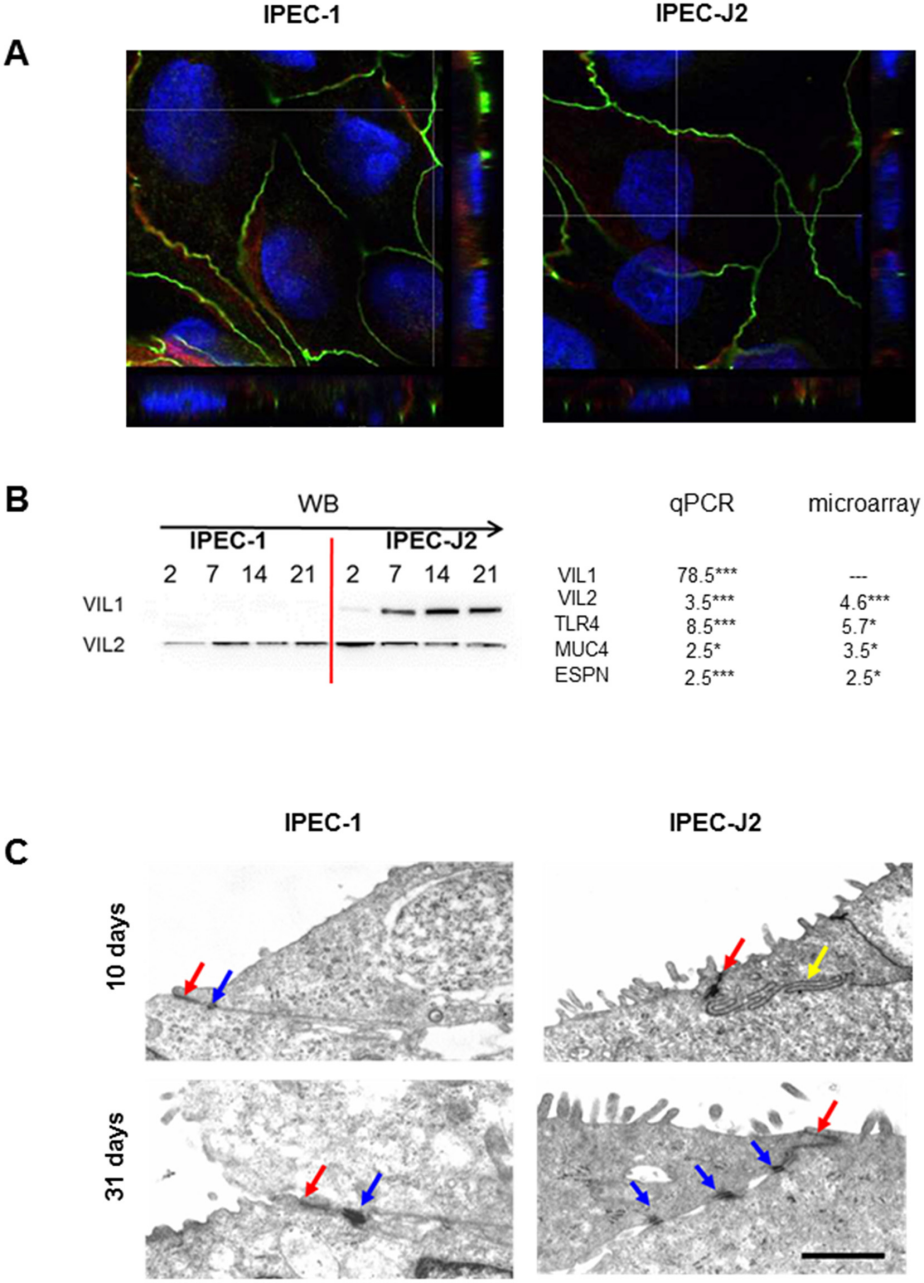
Polarisation of IPEC-1 and IPEC-J2, confocal microscopy and transmission electron microscopy. (A) Ten days old IPEC-1 and IPEC-J2 cells were fixed and stained for ZO-1 (*Zonula occludens*, green) and beta-catenin (*Zonula adherens*, red). Both cells lines showed a polarised structure with ZO-1 immunoreactivity at the apical pole and the underlying beta-catenin expression. (B) Important genes of a polarised brush border were analysed using microarray and qPCR. Here, significant differences were found between the cell lines. VIL1 (villin-1), VIL2 (villin-2 = ezrin), TLR4 (toll like receptor 4), MUC4 (mucin 4) and ESPN (espin) were significant up-regulated in IPEC-J2 in comparison to IPEC-1 in microarray as well as in qPCR. Villin1 and villin2 (= ezrin) were followed up by Western blot analyses. No villin-1 was detected in IPEC-1 but a strong expression in IPEC-J2 over time. Villin-2 was expressed in both cell lines. (C) Cells were cultured for 10 or 31 days and analysed using transmission electronmicroscopy. Both cell lines showed well-developed tight junctions at day 10 and 31 (red arrows). Desmosomes (blue arrows) and interdigitation (yellow arrow) were also observed. No differences were detected concerning microvilli length between both cell lines and at both time points. In contrast, the number of microvilli differed between the cell lines (IPEC-J2>IPEC-1).

IPEC-1 and IPEC-J2 form a continuous monolayer characterised by polarised growth, transepithelial resistance (TEER) and the expression of actin, villin-1 and villin-2 (= ezrin) (Fig 2). The polarised structure was confirmed by immunofluorescence-based detection of ZO-1 and β-catenin (Fig 2A). Three-dimensional reconstruction of optical sections of confocal microscopy showed an apical localisation of ZO-1 (green) and a lateral localisation of β-catenin (red). Both cell lines showed increasing TEER-values with increasing duration of cultivation. On day 10, IPEC-1 and IPEC-J2 exhibited a TEER about 7 kOhm*cm^2^ (IPEC-1: 7.0±1.2 kOhm*cm^2^; IPEC-J2: 7.8±1.0 kOhm*cm^2^).

In the next step, other genes of cytoskeleton proteins that are in close contact to actin and important markers of the brush border were analysed. VIL1 (Villin 1), VIL2 (Villin 2 = Ezrin), TLR4 (Toll like receptor 4), ESPN (Espin; all: p≤0.001) and MUC4 (Mucin 4; p≤0.05; Table 3) were significantly up-regulated in IPEC-J2. The qPCR and microarray showed same results with the exception of VIL1. This gene was unfortunately not included in the gene chip array, but a 78.5 fold increase was detected by the qPCR. Furthermore, morphology of the brush border was analysed by TEM after 10 and 31 days of cultivation (Fig 2C). On day 10, 600 cells per 8 mm length of the basal growth area were detected in both cell lines. On day 31, 660 cells/8 mm were counted for IPEC-1 and 640 cells/8 mm for IPEC-J2. On day 10 of cultivation no differences of the microvilli length of IPEC-1 and IPEC-J2 was observed (IPEC-1: 0.31 μm; IPEC-J2: 0.29 μm) (Fig 2C). The same results were detected after 31 days of cultivation (IPEC-1: 0.27 μm; IPEC-J2: 0.28 μm). However IPEC-J2 showed more microvilli (mv) than IPEC-1 on day 10 and day 31 (day 10: IPEC-1 = 16.1 mv/10 μm; IPEC-J2 = 30.8 mv/10 μm; day 31: IPEC-1 = 15.3 mv/10 μm; IPEC-J2 = 29.9 mv/10 μm).

**Table 3:** Comparison of microarray data and qPCR. Important genes of the significantly regulated pathways shown in Table 2 were analysed via qPCR. The microarry data are shown and two reference genes were used to illustrate significant differences between both cell lines. Asterisks indicate significant differences from IPEC-1.

	Microarray analyses	reference gene 18S	reference gene beta-actin
	IPEC-J2 (IPEC-1 ≙ 1)	IPEC-J2 (IPEC-1 ≙ 1)	IPEC-J2 (IPEC-1 ≙ 1)
PDH	0.7 ***	0.67**	0.6***
SDH	1.4***	1.45***	1.01
claudin 3	1.4	17.71***	14.66***
Villin2	4.6***	4.11***	3.41***
Villin1	---	82.90***	70.52***
Cyc1	0.8*	0.71**	0.62***
TLR4	5.7*	7.46***	6.76***
Muc4	3.5*	2.27**	1.85*
p53	27.04 **	357.94***	278.4***
BAD	1.79***	1.97***	1.59***
BAX	2.23**	1.58***	1.27
BCL-X	---	1.13	0.94
RPL10A	0.9*	0.79*	0.7***
Claudin-1	0.7*	0.73**	0.62**
Claudin-7	2.1**	2.17***	1.93***
HIF1a	1.2*	1.29*	1.01
COX5B	1.23*	1.08	0.86
Occludin	0.6***	0.50***	0.39***
ESPN	2.5*	3.14***	2.46***
18S	---	---	0.89
beta-actin	---	1.12	---

*p≤0.05;

**p≤0.01;

***p≤0.001

**Fig 3:**
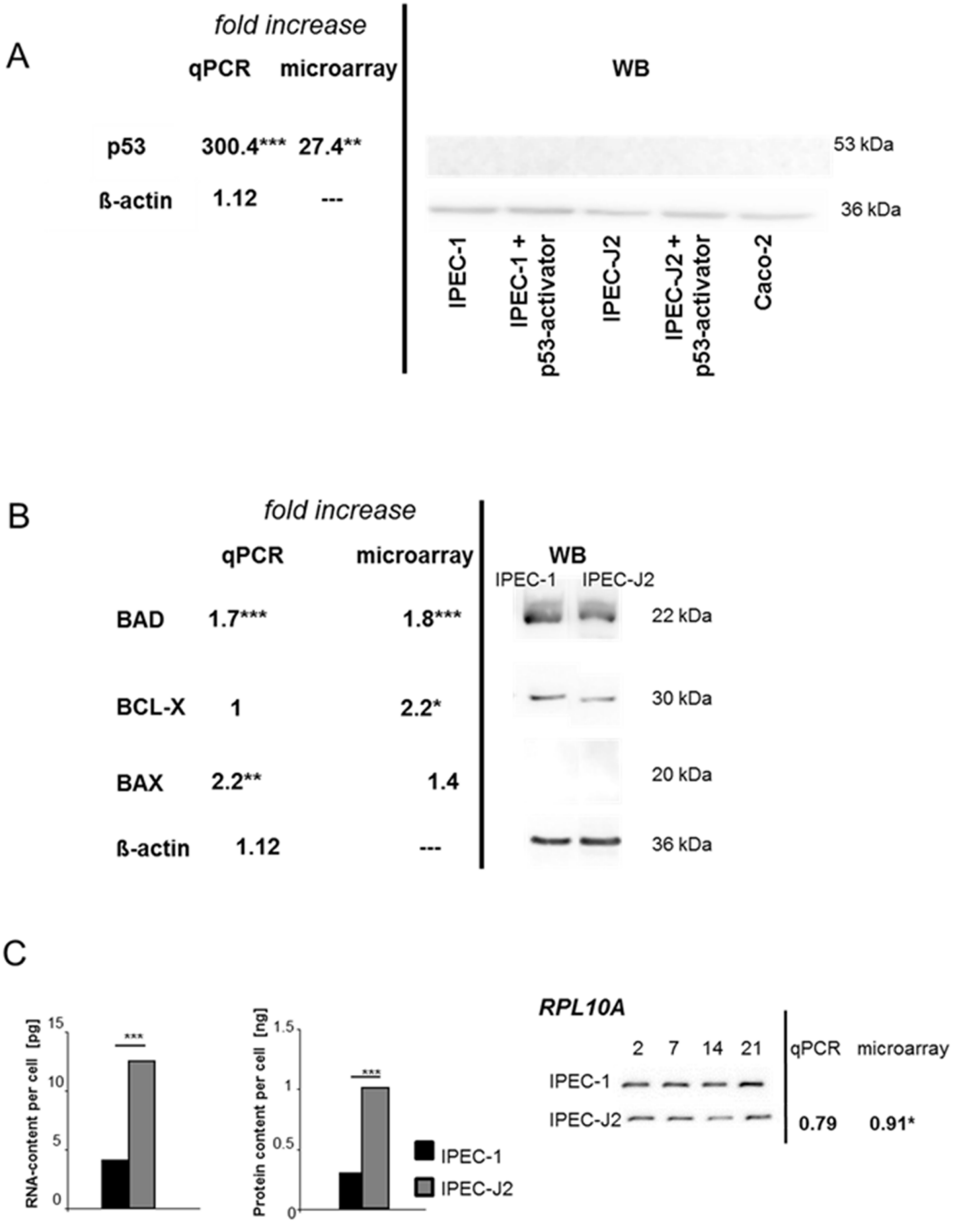
Analysis of cell cycle specific genes on mRNA and protein level. The expression of p53 was analysed by microarray, qPCR and Western blot. (A) IPEC-J2 showed a 27.4 fold increase of the p53-mRNA level in comparison to IPEC-1. This was confirmed by qPCR and a 300-fold increase was detected in IPEC-J2. On the other hand, no protein expression was observed in IPEC-1/IPEC-J2 and Caco-2 as well as in the cells, which were treated with the p53-activator. In the next step, the expression of BAX, BAD and BCL-X was analysed using microarray, qPCR and Western blot. (B) BAD showed a 1.8 fold increase in IPEC-J2 in the microarray. Same results were found in qPCR, but no differences in the protein expression were found between the cell lines. A significant BCL-X up-regulation was detected in the microarray in IPEC-J2 but no differences were present between IPEC-1 and IPEC-J2 in the Western blot analyses. No BAX-protein was observed in both cell lines. When the total RNA and protein content was examined, significant differences were found between the cell lines. IPEC-J2 showed a higher RNA- and protein content in comparison to IPEC-1. RPL10A as important gene of the 60S ribosomal subunit was significantly decreased in the microarray but not in qPCR in IPEC-J2 in comparison to IPEC-1. Western blot analyses showed a slightly decrease of the protein in IPEC-J2 over time. (C) The RNA and protein content per cell was measured in both cell lines. IPEC-J2 showed a significant higher level in RNA and proteins. On the other hand, a slight decrease on protein level of RPL10A was found in IPEC-J2 in comparison to IPEC-1 in Western blot analyses.

The microarray provided many data concerning cellular pathways, as shown in Table 2. In the IPEC-1 and IPEC-J2 cells the pathways in cancer, protein transport and RNA-degradation were of special interest as these pathways reflect on the physiological cell function necessary for a situation comparable to the *in vivo*-intestinal function. Fig 3 data for p53, BAD, BCL-X, BAX and RNA content in comparison to protein content are given.

A significant increase of p53 was detected in IPEC-J2 (p≤0.001) in qPCR analyses in comparison to IPEC-1. Here, we found a 27-fold increase in the microarray analyses and a 300-fold increase in the qPCR (Fig 3A; Table 3). In the next step, p53-protein was examined by Western blot analyses, but no protein was detected in both cell lines. P53-activator was used to introduce p53-protein in the cells but no expression was observed (Fig 3A). On the other hand, the BAD-protein was detected in both cell lines. Other genes of the cell cycle like BCL-2 and BAX showed a significantly 2.2-fold increase in the microarray but not in qPCR (Fig 3B). This was also confirmed by Western blot analyses. We detected the BCL-protein in both cell lines but no BAX-expression was observed (Fig 3B).

The pathway “RNA degradation” was significantly down-regulated in IPEC-J2. Furthermore, we examined the RNA-content per cell and compared both cell lines. IPEC-J2 showed a significant higher RNA-content than IPEC-1 (p<0.001; Fig 3C). This goes along with the 300-fold higher p53-RNA content in IPEC-J2. In the next step, the protein content was analysed as an important indicator for the pathway “ribosome” which is down-regulated in IPEC-J2 in comparison to IPEC-1. Here, 34 genes are significantly affected. 22 genes are involved in the 60S ribosomal proteins (e.g. RPL10-15), 9 genes in the 40S ribosomal proteins (e.g. RPS5-8), 1 gene in the 60S mitochondrial ribosomal protein and 1 is predicted to be a hypothetical protein. The protein content per cell was measured and calculated for both cell lines. IPEC-J2 showed a significant higher protein content than IPEC-1 (p<0.001; Fig 3C). Furthermore, RPL10a, an important gene of the 60S ribosomal proteins, was analysed via qPCR and Western blot analyses (Fig 3C; Table 3). RPL10a showed a significant decrease in the microarray but not in qPCR in IPEC-J2 in comparison to IPEC-1. Furthermore, the RPL10a protein decreased slightly in IPEC-J2 over time.

**Fig 4:**
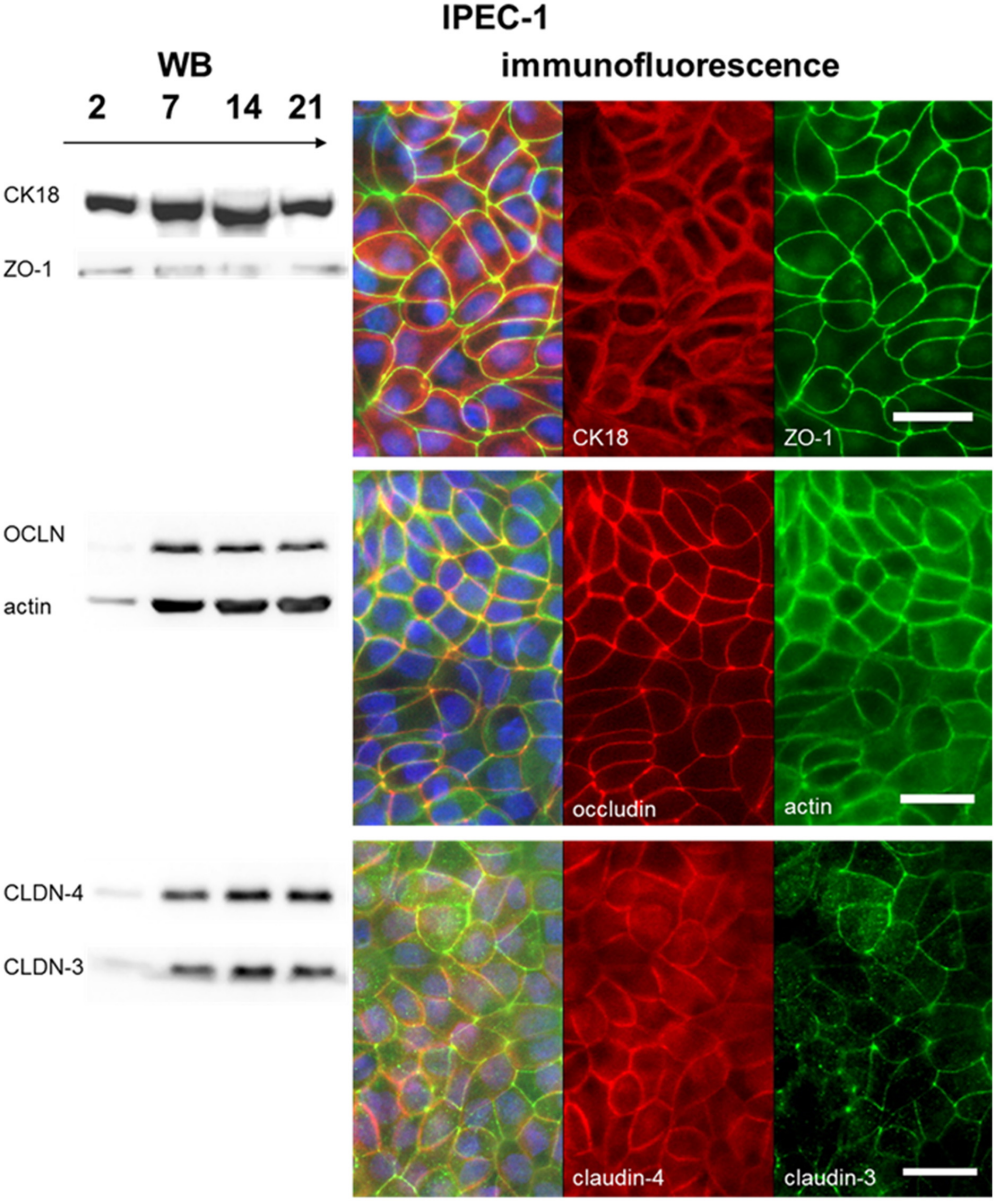
Analysis of the protein expression of tight junctions and cytoskeleton—IPEC-1. Different proteins of the tight junctions and cytoskeleton were analysed by Western blot and immunofluorescence: CK18, β-actin, ZO-1, occludin, claudin-3 and claudin-4. At day 2 of culture IPEC-1 showed in the Western blots a weak protein expression of all proteins studied. A strong ZO-1 and occludin immunoreactivity was found at the border of the cells. In IPEC-1 ZO-1 showed a low expression, whereas a large amount of occludin was present. The cytoskeleton proteins CK18 and actin were strongly expressed at the border of the IPEC-1 cells. Furthermore, no stress fibres were detected in the cells. Claudin-3 and claudin-4 were observed in the cells. A spot like character of claudin-3 distribution in the area where at least three cells were closely located. Claudin-4 showed an expression at the cell border but also within the cytoplasm. (blue = DAPI; bar = 20 μm)

**Fig 5:**
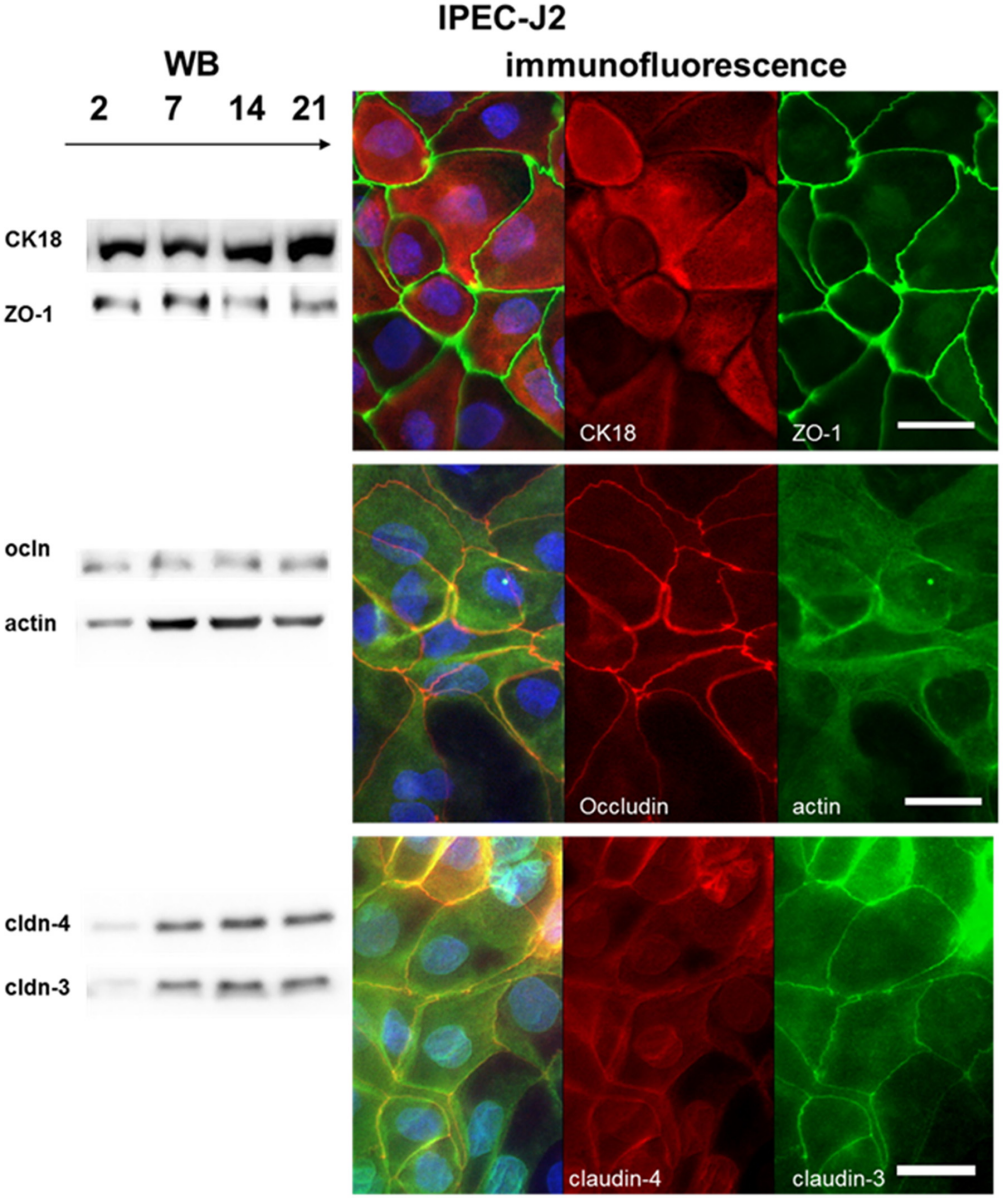
Analysis of the protein expression of tight junctions and cytoskeleton—IPEC-J2. Western blot analyses and immunofluorescence were used to examine different proteins of the tight junctions (ZO-1, occludin, claudin-3 and claudin-4) and the cytoskeleton CK18 and actin). Results comparable to IPEC-1 were found in IPEC-J2, here a weak expression was found at day 2 of culture for all proteins analysed using Western blot. The tight junctions proteins ZO-1 and occludin showed a strong expression at the cell border, however in this cell type ZO-1 had a much higher immunoreactivity as occludin. No actin-stress fibres were found in IPEC-J2. Claudin-3 and claudin-4 were present, but claudin-4 showed higher expression in the cytoplasm than at the cell border in comparison to claudin-3. (blue = DAPI; bar = 20 μm)

A more detailed examination of the morphology of IPEC-1 and IPEC-J2 showed the clear network of the membrane-linked proteins involved in the cell-cell-contacts. 28 genes are regulated in the microarray analyses that are involved in the network of the connected cells. Claudin-1, claudin-3, claudin-7 and occludin were chosen as marker for cell-cell contacts and tight junctions. An increased mRNA-level of claudin-3 and claudin-7 (both: p≤0.001, Table 3) was detected in IPEC-J2. In comparison to IPEC-1, a decreased mRNA-level of claudin-1 and occludin (both: p≤0.001) was noticed. These data were obtained by microarray data. Differences between the microarray and the qPCR were observed in claudin-3, which was significantly up-regulated in the qPCR but not in the microarray. IPEC-1 and IPEC-J2 were cultured on membranes and analysed at different time points: day 2, 7, 14 and 21 (Figs 4 and 5) via Western blot. ZO-1 and occludin showed opposed characteristics: ZO-1 < occludin in IPEC-1 and ZO-1 > occludin in IPEC-J2. In both cell lines, a strong expression of claudin-3 and claudin-4 was detected at day 7, 14 and 21 of culture by Western blot analyses. Claudin-3 and claudin-4 were observed in the cells. Claudin-4 showed higher expression in the cytoplasm than at the cell border in comparison to claudin-3 in IPEC-J2.

In comparison to IPEC-J2 cells, the distribution of claudin-3 is in IPEC-1 more spot-like in the region where three cells have a contact area (Figs 4 and 5). Morphological differences were also found, namely villin and actin increased in IPEC-J2 in the immunofluorescence (Fig 5). No differences in beta-actin expression were detected by microarray, qPCR and Western blot analyses (Table 3).

**Fig 6:**
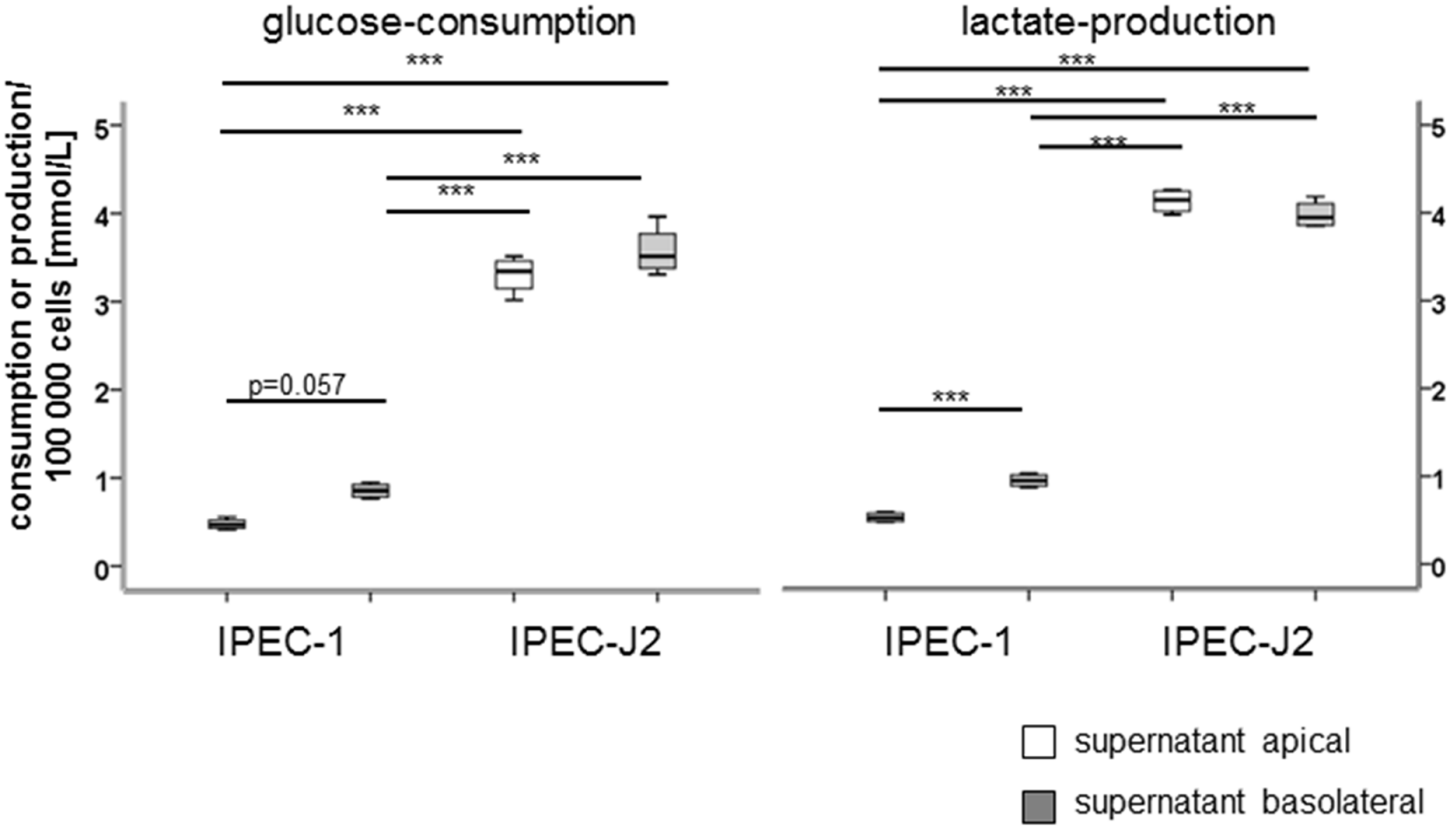
Glucose consumption and lactate production in IPEC-1 and IPEC-J2. Apical and basolateral supernatants of cells cultured on membranes were collected and analysed using the Roche/Hitachi Cobas c system. IPEC-J2 showed a significant higher apical and basolateral glucose consumption in comparison to IPEC-1 (*apical*: IPEC-1: 0.48 μmol/100000 cells, IPEC-J2: 3.3 μmol/100000 cells; *basolateral*: IPEC-1: 0.95 μmol/100000 cells, IPEC-J2: 3.57 μmol/100000 cells). Comparable results were found with the focus on lactate production (*apical*: IPEC-1: 0.48 μmol/100000 cells, IPEC-J2: 4.1 μmol/100000 cells; *basolateral*: IPEC-1: 0.95 μmol/100000 cells, IPEC-J2: 3.98 μmol/100000 cells).

**Fig 7:**
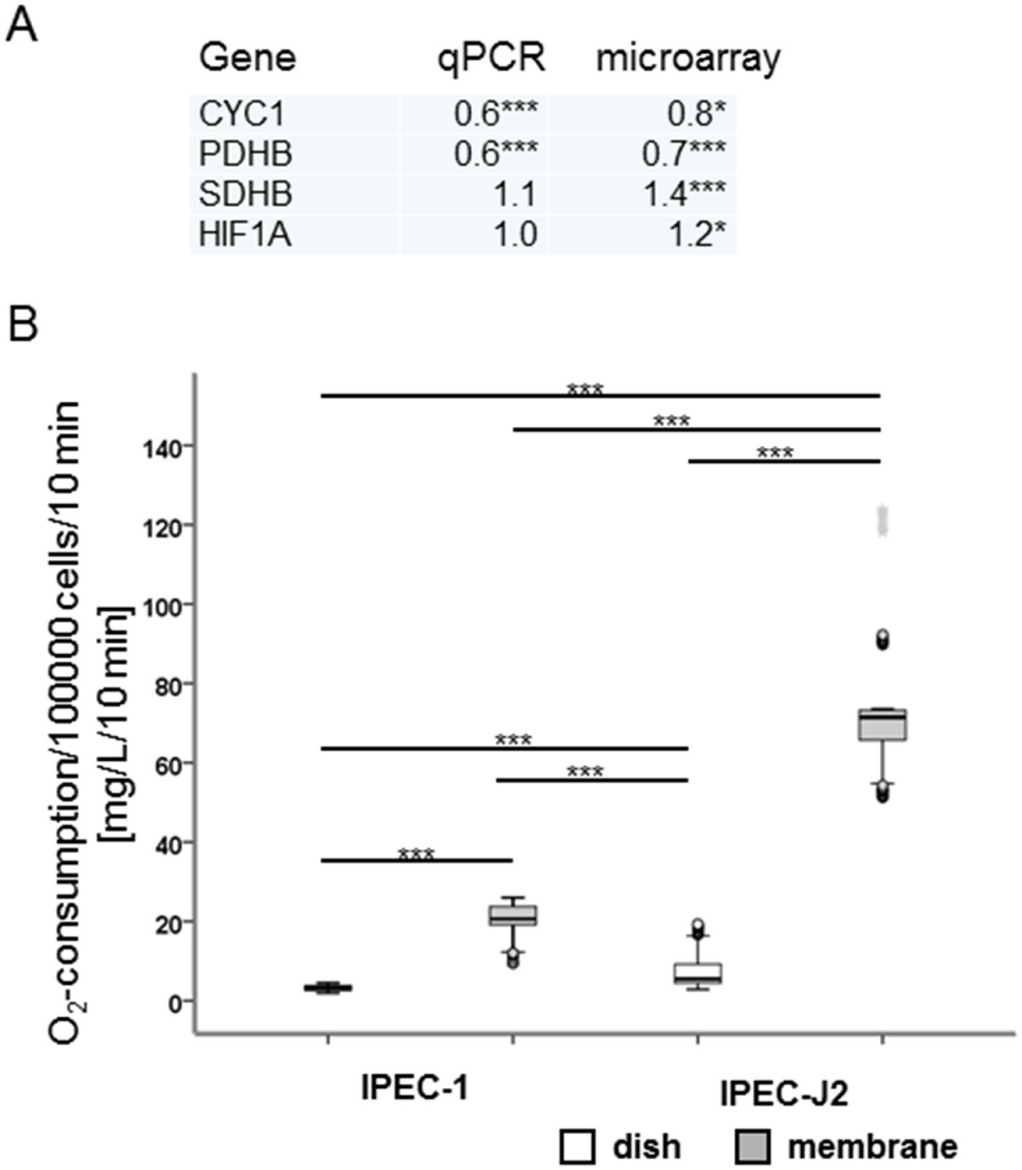
Analyses of important genes of the metabolism and oxygen-consumption. Important genes of the metabolism were analysed in both cell lines cultured on membranes for 10 days using microarray analyses and qPCR (A). PDHB (pyruvate dehydrognase subunit B) and CYC1 (cyctochrome C) are significantly down-regulated in the microarray and qPCR. SDH (succinate dehydrogenase subunit B) and HIF1a (hypoxia inducible factor 1a) are both significantly up-regulated in the microarray but not in qPCR. Furthermore, oxygen-consumption of both cell lines cultured on dishes or membranes for 10 days was examined. Both cell lines showed a significant higher O_2_-consumption on membranes (IPEC-1: 20.07 nmol/100 000 cells; IPEC-J2: 75.27 nmol/100 000 cells) in comparison to dishes (IPEC-1: 3.18 nmol/100 000 cells; IPEC-J2: 8.18 nmol/100 000 cells). At the same time, a significant higher oxygen-consumption was found in IPEC-J2 in comparison to IPEC1, which was independent of the culture support.

**Fig 8:**
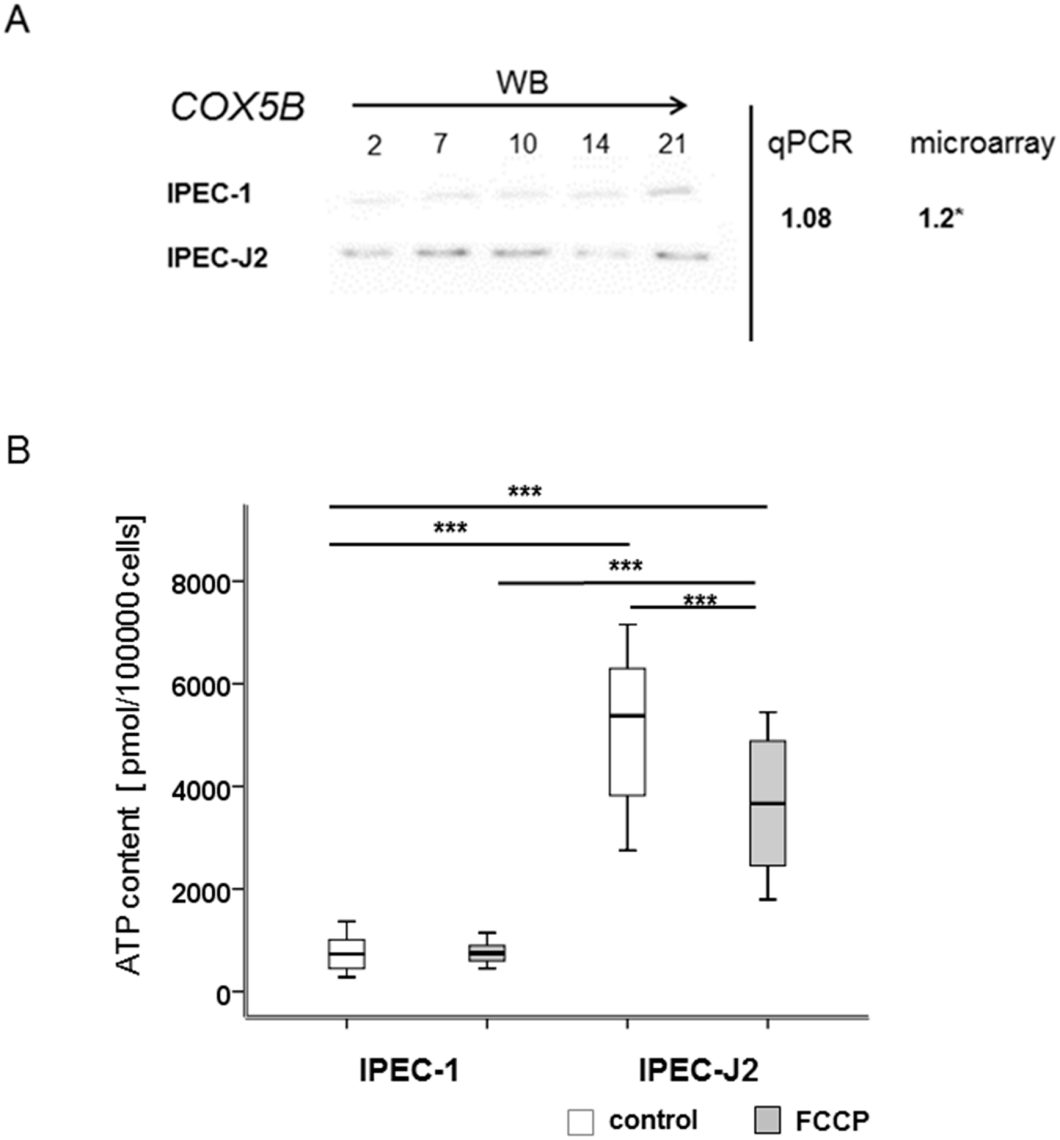
Cytochrome C-oxidase and ATP-production. COX5B was analysed by microarray analyses and qPCR. IPEC-J2 cells showed a significantly increased RNA level of COX5B in the microarray (Fig 8A; p<0.05) but not in qPCR in comparison to IPEC-1. Furthermore, IPEC-J2 showed a higher protein content of COX5B than IPEC-1. In the next step, cells were analysed using an ATP Bioluminescence Assay Kit CLS II (Fig 8B; Roche, Germany). Carbonylcyanid-p-trifluoromethoxyphenylhydrazon (FCCP) was used as control. IPEC-1 (control: 749.39 pmol/100 000 cells; FCCP: 745.31 pmol/100 000 cells) showed no reaction after the application of FCCP in comparison to IPEC-J2. IPEC-J2 showed a significant higher ATP content in comparison to IPEC-1 (p<0.001; IPEC-1: 749.31 pmol/100 000 cells; IPEC-J2: 5104.17 pmol/100 000 cells). Furthermore, a significant decrease in the ATP-content was found in IPEC-J2 after the application of FCCP (p<0.001; IPEC-J2-FCCP: 3680 pmol/100 000 cells).

To a better understanding of the metabolism of IPEC cells, glucose utilisation, lactate production, oxygen consumption and ATP-content were examined (Figs 6, 7 and 8). In Fig 6 the results for the glucose-consumption and lactate-production in both cell lines are shown. Differences were found between the cell lines. IPEC-J2 showed a higher glucose-consumption than IPEC-1 (*apical*: IPEC-1: 0.48 μmol/100000 cells, IPEC-J2: 3.3 μmol/100000 cells; *basolateral*: IPEC-1: 0.95 μmol/100000 cells, IPEC-J2: 3.57 μmol/100000 cells). Similar results were found with the focus on lactate-production (*apical*: IPEC-1: 0.48 μmol/100000 cells, IPEC-J2: 4.1 μmol/100000 cells; *basolateral*: IPEC-1: 0.95 μmol/100000 cells, IPEC-J2: 3.98 μmol/100000 cells). Furthermore, the microarray analyses showed 29 significantly regulated genes like COX5B, COX7A2, COX7C, COX8A, ATP5D, ATP5E, ATPH, NDUFA2, NDUFA4 and NDUFB2. In addition, important genes of the aerobic metabolism like CYC1, PDHB and SDHB were examined (Fig 7A). CYC 1 (cytochrome C) plays an important role in the mitochondrial respiratory chain by transferring electrons from the Rieske iron-sulfur protein to cytochrome C and is significant down-regulated in IPEC-J2 in comparison to IPEC-1 in qPCR and in the microarray. PDHB and SDHB showed opposed characteristics. Pyruvate dehydrogenase subunit B (PDHB) is a nuclear encoded mitochondrial multi-enzyme complex that catalyses the conversion of pyruvate to acetyl-CoA and carbon dioxide, it is significantly down-regulated in IPEC-J2 in comparison to IPEC-1 and. On the other hand, succinate dehydrogenase subunit B (SDHB) is significantly up-regulated and is involved in complex II and known as iron-sulfur subunit. It catalyses the oxidation of succinate. Hypoxia inducible factor (HIF1a) plays an essential role in cellular and systemic responses to hypoxia and is significantly higher expressed in IPEC-J2 in the microarray but not in qPCR. In the next step, oxygen-utilisation was examined (Fig 7B). In both cell lines, the use of a membrane growth support significantly increases the oxygen utilisation—demonstrating the membrane grown cells to be a more physiological cell culture system. In addition, IPEC-J2 (dish: 8.18 nmol/100 000 cells; membrane: 75.27 nmol/100 000 cells) showed on dishes as well as on membranes a significantly higher oxygen-consumption than IPEC-1 (dish: 3.18 nmol/100 000 cells; membrane: 20.07 nmol/100 000 cells). One important gene of the complex IV of the respiratory chain is COX5B. COX5B was analysed by microarray, qPCR and Western blot analyses. IPEC-J2 cells showed a significantly increased RNA level in the microarray but not in qPCR in comparison to IPEC-1 but a higher COX5B-protein content was observed in IPEC-J2 (Fig 8A).

As the metabolism of the epithelial cells is mainly linked to a physiological role of the mitochondria (complex V of the respiratory chain), the decoupling of the respiratory chain was performed using Carbonylcyanid-p-trifluoromethoxyphenylhydrazon (FCCP). The results in ATP-production are given in Fig 8. IPEC-J2 (control: 5104.17 pmol/100 000 cells; FCCP: 3680 pmol/100 000) showed a 7-fold increased ATP-level per 100 000 cells than IPEC-1 (p<0.0001; Fig 8). FCCP resulted in a significant decreased ATP-content in IPEC-J2 but not in IPEC-1 (control: 749.39 pmol/100 000 cells; FCCP: 745.31 pmol/100 000 cells).

## Discussion

Only a few non-transformed intestinal cell lines such as IEC-6 and IEC-18 exist [14]. In 1989, IPEC-1 and IPEC-J2 were established from 12-hours old piglets and described as non-transformed [9; 15]. Therefore, they are a convincing model for studying principal intestinal mechanisms like transport of nutrients, toxins [16], differentiation of intestinal epithelial cells and metabolism of these cells [17]. Both cell lines show a different morphology—IPEC-1 a cobblestone and IPEC-J2 an elongated phenotype with a higher cell area [13]. In 1964, MacPherson and Montagnier [18] introduced the anchorage independent cell growth and clone formation in soft-agar as an *in vitro* test of cancer. Normal epithelial cells are supported by a basement membrane, which provides survival and proliferative signals, and they undergo apoptosis when seeded in suspension culture [19]. In contrast to the carcinoma derived Caco-2 cells, neither IPEC-1 nor IPEC-J2 exhibit typical anchorage independent growth that strongly indicates the non-carcinoma nature of the cell lines. Caco-2 cells are able to evade the attachment-regulated apoptosis, which is called anoikis and this leads to uncontrolled proliferation [20].

Furthermore, the absorptive epithelium of the gastrointestinal tract developed the brush border, a highly specialised apical membrane that increases the surface area. A well-developed brush border is an important marker of polarised intestinal epithelial cells. TEM analyses at different time points showed a two-fold higher number of microvilli in IPEC-J2 than in IPEC-1 at both time points (day 9: IPEC-1 = 16.1; IPEC-J2 = 30.8; day 31: IPEC-1 = 15.3; IPEC-J2 = 29.9). These microvilli are primarily composed of six proteins: actin, fimbrin, villin, brush border myosin (Myo1A), calmodulin and a non-erythrocytic spectrin [21]. This structure corresponds to our microarray data with the focus on the significant up-regulation of the pathway “actin-cytoskeleton”. The microvilli core bundle of about 19 actin filaments are cross-linked by villin and fimbrin [22]. Interestingly, in our study villin (VIL1) was significantly up-regulated in IPEC-J2, an indication that in this cell line the microvilli are more efficiently cross-linked. No villin-protein but microvilli were detected in IPEC-1 when IPEC-1 cells were seeded on dishes and membranes [13]. We suggest that villin is not necessary for the bundling of actin filaments but the more efficient composition including villin may result in higher numbers of microvilli as observed in IPEC-J2.

Many cancer cell lines like Caco-2 and HT-29 also express high level of villin because of their ability to develop an enterocytic differentiation [3]. We had already mentioned that IPEC-1 and IPEC-J2 are non-transformed and have their origin not in a carcinoma. In contrast, we observed a significant up-regulation of the ‘pathways in cancer’ in IPEC-J2 with P53 as the most important gene, but another marked function of this gene is the role in regulating cell growth and the differentiation through up- and down-regulating different genes like LIG1, MCL-1, P21, BAD, BAX and BCL-2 [23]. The induction of apoptosis is regulated by p53 through the activation of the BAX gene expression, BCL-2 is changed and cells undergo apoptosis [24]. In our study, the ratios of BAX/BCL-2-mRNA are similar and a strong expression of BCL but no BAX-protein expression was observed in both cell lines. Low or weak BAX-expression is correlated with prolonged cell survival and an enhanced resistance to CD95-mediated or serum-starvation-induced apoptosis [25]. Transfection experiments have shown that p53-expression can down-regulate BCL-expression and can up-regulate BAX-expression [26]. Therefore, Miyashita and co-workers postulated that p53 and BCL are inversely related [26]. In our study, we observed an inverse relationship of P53 and BCL as well. Therefore our data provide a putative mechanism for the longevity of both cell lines. P53 is also responsible for an increased transcription and expression of BAD [27], which is in this study significantly increased in the mRNA-level in IPEC-J2 and a strong BAD-expression was detectable in Western blot analysis. BAD is also able to direct p53 to mitochondria and forms there a bad/p53 complex [27]. Jiang et al. showed an accumulation of bad and p53 in the mitochondria and a reduced occurrence in the cytoplasm, which was associated with a release of cytochrome C [27].

Differences between the cell lines were found in the tight junctions (down-regulated in IPEC-J2). *Zonula occludens* (tight junctions), *Zonula adhaerens* and the basally localised desmosomes are commonly found between epithelial cells *in vivo*. Furthermore, *Zonula adhaerens* and *Zonula occludens* serve as structurally landmark for cell polarisation due to the defining of an apical/basolateral axis [28]. Proteins of *Zonula occludens* regulate barrier function [29]. In this context the significant decrease of the mRNA-level of claudin-1 and occludin as well as a significant increase of the mRNA-level of claudin-3 and claudin-7 in IPEC-J2 we observed is of major interest. At different time point claudin-3, claudin-4, ZO-1 and occludin were detectable in IPEC-1 and IPEC-J2 cells by Western blot. We found a higher degree of claudin-4 in the cytoplasm of IPEC-J2 than at the border of the cells. Furthermore, we observed a higher protein level of occludin in IPEC-1 in comparison to IPEC-J2 using Western blot analyses and also in the immunofluorescence. This is in accordance with the mRNA-level in both cell lines.

Furthermore, differences between IPEC-1 and IPEC-J2 were found in two important pathways of cell metabolism namely oxidative phosphorylation and glycolysis. GAPDH, HIF1a and PDH are important genes, which are up-regulated when cells produce ATP via glycolysis. HIF1a is the most important, because it regulates all other genes that are involved in the glycolysis. In our study, we did not detect any differences between the cell lines in HIF-1a-mRNA in the qPCR but an up-regulation in the microarray in IPEC-J2 (Fig 7A). In a recent study it was demonstrated that an enhanced oxygen supply triggers the structural and functional differentiation in an air-liquid interface culture in IPEC-1 and IPEC-J2 [13]. In the present study we found differences between the cell lines due to the improved oxygen supply. Therefore, the present results further support a differentially regulated metabolism of the cells. In IPEC-J2, oxidative phosphorylation is significantly up-regulated (Table 2). Functional analyses were examined via ATP-measurement, O_2_- and glucose-consumption and lactate-production. IPEC-J2 showed a significantly higher ATP-content than IPEC-1. FCCP results in a significantly decreased ATP-content in IPEC-J2 but not in IPEC-1. These results go along with the O_2_-utilisation. IPEC-J2 showed a significant higher O_2_-consumption than IPEC-1 on dishes as well as on membranes. Furthermore, glucose-consumption and lactate-production were examined. A significant higher glucose-consumption and lactate-production were found in IPEC-J2 in comparison to IPEC-1. We suppose that IPEC-J2 used aerobic as well as anaerobic glycolysis to produce ATP due to: 1. high ATP content (aerobic), 2. FCCP results in significantly decreased ATP-levels, 3. high glucose consumption (anaerobic), 4. high O_2_-consumption (aerobic) and 5. high lactate production (anaerobic). IPEC-1 seems to be in a “steady state” or non-proliferative state because: 1. low levels of ATP, 2. low O_2_ consumption, 3. low glucose consumption and 4. low lactate production [30]. Glucose metabolism in cell culture is affected by glucose concentration, temperature and pH-value of the medium [31], which leads to the hypothesis that there are major differences in the glucose metabolism in both cell lines.

## Conclusion

In conclusion we demonstrate that both cell lines are differentially regulated in: A. Cytoskeleton with effects on the brush border, B. Tight junctions with no effect on TEER values but possible on ion-transport; C. Metabolism with the focus on O_2_-consumption, glucose-utilisation and lactate production with a significant effect on ATP-production. When designing experiments using epithelial cell lines the present results should be taken into account, especially when analysing the metabolism of the cells, e.g. in terms of consumption of glucose and ATP.
